# Synergistic inhibition of pathogenic fungi by oleanolic acid combined with azoles

**DOI:** 10.1128/spectrum.00854-25

**Published:** 2025-07-07

**Authors:** Tian Chen, Shaolan Liu, Fei Yang, Zhangxuan He, Menghua Tang, Lu Ge, Hongyi Zhang, Sijie Liu, Xiaolei Zhu, Mengqi Peng, Heng Zhang, Wenxu Cheng, Yi Sun

**Affiliations:** 1 Department of Clinical Medicine, Yangtze University 47897, Jingzhou, Hubei, China; 2 Department of Dermatology, Hubei Provincial Clinical Research Center for Diagnosis and Therapeutics of Pathogenic Fungal Infection, Jingzhou Hospital Affiliated to Yangtze University, Jingzhou, Hubei, China; 3 Department of Otolaryngology, Jingzhou Hospital Affiliated to Yangtze University, Jingzhou, Hubei, China; Universidade de Sao Paulo, Ribeirao Preto, Sao Paulo, Brazil

**Keywords:** oleanolic acid, azoles, synergism, antifungal activity, pathogenic fungi

## Abstract

Invasive fungal infections and emerging antifungal resistance mediated by pathogenic fungi, notably *Candida* spp., *Aspergillus* spp., *Cryptococcus neoformans*, and *Exophiala dermatitidis,* pose a critical challenge to global public health systems. This study evaluated the synergistic antifungal activity of oleanolic acid (OA) combined with five azole agents (itraconazole [ITR], voriconazole [VOR], posaconazole [POS], isavuconazole [ISA], and fluconazole) against *Candida* spp., *Aspergillus* spp., *E. dermatitidis*, and *C. neoformans*. Using CLSI-compliant broth microdilution assays (M27-A3/M38-A2), we determined the minimum inhibitory concentrations (MICs) of oleanolic acid against four fungal pathogens and evaluated *in vitro* synergistic interactions with five azoles to assess their therapeutic potential. OA exhibited no intrinsic fungicidal activity when administered alone. However, synergistic inhibition was observed in specific azole combinations across tested pathogens: *Candida* spp. (*n* = 18) demonstrated 33% (6/18) and 56% (10/18) synergy rates with ITR and POS, respectively. *Aspergillus* spp. (*n* = 30) showed enhanced synergism at 80% (24/30) for ITR and 97% (29/30) for POS. All OA-azole combinations were synergistic against *C. neoformans* (*n* = 8), with rates of 75% (6/8) for ITR, 75% (6/8) for VOR, 87.5% (7/8) for ISA, and 100% (8/8) for POS. In contrast, *E. dermatitidis* (*n* = 21) responded only to OA/POS (52%, 11/21). Overall synergistic frequencies were POS (75%, 58/77) > ITR (47%, 36/77) > ISA (9%, 7/77) > VOR (8%, 6/77). The OA-azole combinations demonstrated significant reductions in azole MIC values and exhibited synergistic antifungal activity, particularly against *Aspergillus* spp. and *C. neoformans*. While the mechanistic basis of OA’s chemosensitization remains uncharacterized, its multi-target pharmacological profile underscores potential utility in combating antifungal resistance.

**IMPORTANCE:**

Invasive fungal infections and antifungal resistance are significant global health challenges. This study highlights the potential of oleanolic acid (OA) combined with azoles to combat these issues. OA alone had no antifungal activity, but its combinations with azoles exhibited notable synergy against major pathogens, particularly *Aspergillus* spp. and *Cryptococcus neoformans*. Notably, OA combined with posaconazole showed synergy against *Exophiala dermatitidis* for the first time. These findings suggest that OA could enhance the efficacy of existing antifungal agents, reduce their required doses, and potentially overcome resistance. This research underscores the importance of exploring OA as a novel adjuvant to improve antifungal therapy.

## INTRODUCTION

Pathogenic fungi, as defined by their ability to cause human disease, represent a critical global health challenge. Collectively, they are responsible for over 1 billion infections annually, spanning from superficial mycoses to life-threatening invasive diseases (1). Among numerous pathogenic fungi, *Aspergillus, Candida, and Cryptococcus neoformans* have attracted significant attention due to their high prevalence and drug resistance, while Exophiala dermatitidis is considered the most virulent species capable of causing disseminated infections (2, 3). These opportunistic pathogens are widely distributed in nature and account for a remarkably high proportion among the fungi causing invasive diseases, thereby posing significant clinical hazards (4).

Among them, *Candida* spp. are clinically significant opportunistic pathogens. They can colonize human mucosal surfaces such as the oral cavity, intestinal tract, and vaginal epithelium, causing invasive candidiasis, including oropharyngeal candidiasis and vulvovaginitis (5). *Aspergillus* spp., with *Aspergillus fumigatus* being the predominant pathogenic species, are ubiquitously distributed in soil and air. They are responsible for invasive aspergillosis with pulmonary and cutaneous manifestations (6). *Cryptococcus neoformans*, a neurotropic pathogen endemic to avian guano-enriched soils across tropical and temperate regions, frequently induces meningoencephalitis and pulmonary infections (7). *Exophiala dermatitidis*, a saprophytic fungus inhabiting decaying organic matter, primarily causes cutaneous phaeohyphomycosis with rare systemic dissemination (8). Immunocompromised individuals, particularly those with advanced HIV infection (AIDS), are high-risk populations for these invasive mycoses (9, 10). Moreover, emerging antifungal resistance in *Aspergillus* spp., *Candida* spp., and *C. neoformans*, driven by distinct virulence mechanisms, poses a significant challenge to contemporary clinical management (11).

Current clinical management of these infections primarily utilizes four classes of antifungal agents: azoles (e.g., itraconazole [ITR], voriconazole [VOR], posaconazole [POS], and isavuconazole [ISA]), polyenes, echinocandins, and the pyrimidine analog 5-fluorocytosine (12). However, escalating antifungal resistance has significantly compromised therapeutic efficacy, necessitating urgent development of alternative or adjunctive agents (12). Combination therapies offer strategic advantages, including enhanced efficacy, reduced dosage requirements, and mitigation of resistance mechanisms (13). The inherent limitations of single-target antifungal agents, which are vulnerable to resistance through mutagenesis-driven mechanisms, have prompted the exploration of multi-target therapeutics as a strategic approach to counter evolving antifungal resistance (14). Prior investigations have validated this combinatorial paradigm through promising natural product-azole pairings, including bis-cinnamamide polyamines as antimicrobial potentiators (15), CaERG6 inhibitors enhancing azole efficacy against *Candida albicans* (16), and the combination of proton-pump inhibitors (e.g., omeprazole) with commonly used azole drugs (e.g., fluconazole [FLU] and ITR) (17).

Oleanolic acid (OA), a naturally occurring pentacyclic triterpenoid, has recently emerged as a promising candidate for combating drug-resistant fungal and bacterial pathogens (18). OA demonstrates broad-spectrum inhibitory activity against diverse fungal strains, including azole-resistant variants, thereby positioning it as a potential adjunct to existing antifungal therapies (19, 20). Preliminary evidence suggests that OA’s mechanism involves dual targeting of fungal cell membrane integrity and intracellular signaling cascades, though precise molecular interactions require further elucidation (21). Despite these promising aspects, the effectiveness of OA in combination with azoles against fungi is not yet clearly evident, and there is a dearth of comprehensive understanding of its synergistic potential with azoles in a clinical context.

To address the critical gap in understanding combinatorial antifungal strategies against resistant pathogens, this study systematically evaluated the *in vitro* synergism between OA and four clinically relevant triazoles—ITR, VOR, POS, and ISA—against 77 clinical isolates of *Aspergillus* spp., *Candida* spp.*, E. dermatitidis,* and *C. neoformans*. For *Candida* testing, FLU substituted ISA to align with species-specific resistance profiles. Using CLSI-compliant broth microdilution assays (M27-A3/M38-A2), minimum inhibitory concentrations (MICs) and fractional inhibitory concentration indices (FICIs) were determined to quantify synergistic interactions.

## MATERIALS AND METHODS

### Fungal strains

A total of 77 clinical fungal isolates were comprehensively evaluated. This collection consisted of 30 *Aspergillus* spp., 18 *Candida* spp., 21 *E. dermatitidis*, and 8 *C*. *neoformans*. Broth microdilution assays were performed in 96-well plates following CLSI guidelines, with quality control strains *Aspergillus flavus* ATCC 204304 and *Candida parapsilosis* ATCC 22019. All isolates were clinically derived from Jingzhou Central Hospital (Hubei Province, China) and the Hubei Provincial Clinical Research Center for Pathogenic Fungal Infection Diagnosis and Treatment (17). All fungal strains were identified by microscopic morphology and molecular sequencing of the internal transcribed spacer (ITS) ribosomal DNA (22). All the sequences of the analyzed strains were uploaded to GenBank (PP069948–PP070390).

### Antifungals and chemical agents

The antifungal agents, including ITR (No. I129771, purity ≥98%), VOR (No. V129745, ≥98%), POS (No. P125008, ≥ 99%), ISA (No. I337027, ≥98%), FLU (No. E129360, ≥98%), and OA (No. O110087, ≥98%), were procured as analytical-grade powders from Shanghai Aladdin Biochemical Technology Co., Ltd. (China). Working concentrations ranged from 0.03 to 8 μg/mL for ITR, VOR, and ISA; 0.015–4 μg/mL for POS; and 0.0625–8 μg/mL for OA. Stock solutions (6,400 µg/mL) were prepared in dimethyl sulfoxide (≥99.9%) and diluted in RPMI-1640 medium buffered with 0.165 M MOPS (pH 7.0).

### Testing the *in vitro* synergy of OA and azoles

Conidia were harvested from *Candida* spp., *C. neoformans*, *Aspergillus* spp., and *E. dermatitidis* cultures grown on Sabouraud Dextrose Agar (SDA) for 2 days (*Candida* spp. and *C. neoformans*), 3 days (*Aspergillus* spp.), and 5 days (*E. dermatitidis*). Conidial suspensions were prepared in 0.9% saline and standardized to final concentrations of 1–5 × 10^6^ CFU/mL for *Candida* spp. and *C. neoformans*, and 1–5 × 10^7^ CFU/mL for *Aspergillus* spp. and *E. dermatitidis*, using a hemocytometer.

Antifungal susceptibility testing was performed according to the Clinical and Laboratory Standards Institute guidelines M27-A3 and M38-A2, referencing established protocols (22). Fungal suspensions of *Candida* spp. and *C. neoformans* were adjusted to 2–4 × 10^3^ CFU/mL, while *Aspergillus* spp. and *E. dermatitidis* were adjusted to 1–3 × 10^4^ CFU/mL in RPMI-1640 medium. Using a checkerboard microdilution method, 50 µL of serially diluted OA was inoculated horizontally, and 50 µL of serially diluted azoles was inoculated vertically into 96-well plates containing 100 µL of fungal suspension. Incubation conditions were standardized at 35°C for 24 hours (*Candida* spp. and *C. neoformans*), 48 hours (*Aspergillus* spp.), and 72 hours (*E. dermatitidis*). Combinatorial interactions between OA and azoles were categorized according to the FICI, which was calculated by the following formula: FICI = (Ac/Aa) + (Bc/Ba), where Ac and Bc are the MICs of the combination antifungal drugs, and Aa and Ba are the MICs of the individual antifungal drugs A and B (23). An FICI of ≤0.5 indicates synergy, an FICI of >0.5 to ≤4 indicates no interaction (indifference), and an FICI of >4 indicates antagonism (24). All tests were performed in triplicate.

### Strain identification

The cultured fungal specimens were taken and preliminarily identified according to their morphological characteristics. Fungal DNA was extracted by the MolPure Fungal DNA Kit, and the ribosomal DNA transcriptional spacer ITS, beta-tubulin, and calmodulin genes were subsequently amplified (Table 1) (17, 22, 25, 26). PCR was performed using the following parameters: 3 min at 95°C, followed by 35 steps of 1 min at 95°C, 1 min at 58.5°C, and 1 min at 72°C, and then a final 10 min at 72°C. The final products were sequenced by Biocompany (BioEngineering [Shanghai] Co., Ltd), and finally, the sequence was blasted in NCBI GenBank. The definitive identification of the *Aspergillus* isolates was accomplished by comparing the sequences with relevant reference sequences in GenBank using the nucleotide BLAST system (https://blast.ncbi.nlm.nih.gov/Blast.cgi).

**TABLE 1:** Primer sets and corresponding amplification targets

Target gene	Primer	Primer DNA sequence (5′−3′)
ITS	ITS1	TCCGTAGGTGAACCTGCGG
	ITS4	TCCTCCGCTTATTGATATGC
Calmodulin	cmd5	CCGAGTACAAGGAGGCCTTC
	cmd6	CCGATAGAGGTCATAACGTGG
Beta-tubulin	Bt2a	GGTAACCAAATCGGTGCTGCTTTC
	Bt2b	ACCCTCAGTGTAGTGACCCTTGGC

## RESULTS

### *In vitro* interactions between OA and azoles against *Aspergillus* spp.

**Fig 1:**
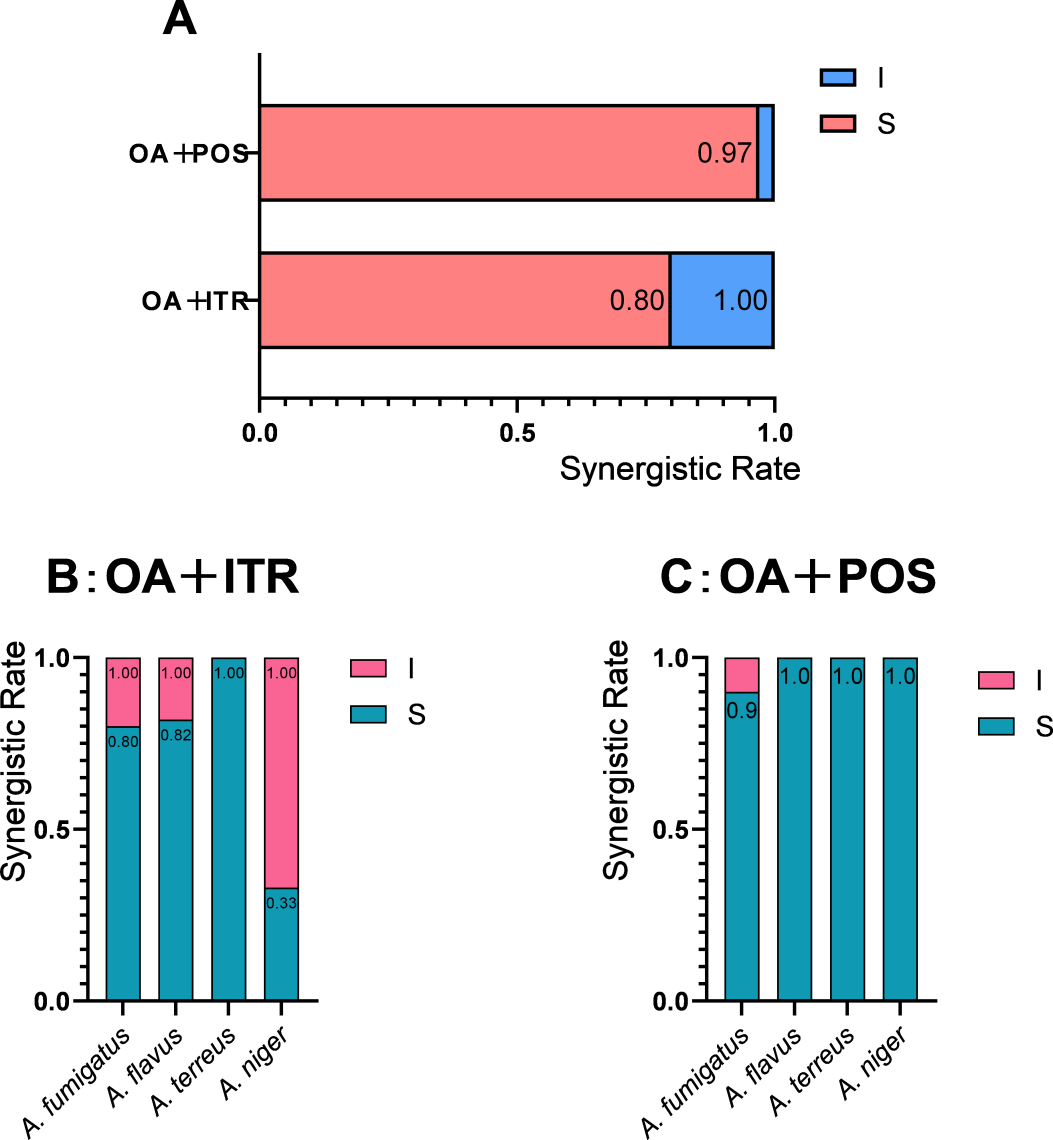
The synergistic rate of OA combined with azoles against *Aspergillus* spp. (A) Interaction profile of OA combined with ITR and POS against *Aspergillus* spp.; (B) OA/ITR interaction profiles across *Aspergillus* species; (C) OA/POS interaction profiles across *Aspergillus* species. S, synergy (FICI ≤ 0.5); I, indifference (FICI from >0.5 to ≤4). The synergism rate was calculated by dividing the number of strains exhibiting synergism by the total number of strains tested.

Used in isolation, OA had no antifungal action against *Aspergillus* spp. In synergistic pairings, the MIC ranges of ITR and POS alone were 2–16 and 1–2 µg/mL, respectively (Table 2). When combined with OA, the MICs decreased for ITR to 0.25–2 µg/mL (eightfold reduction max) and for POS to 0.063–0.25 µg/mL (32-fold reduction max). A total of 30 *Aspergillus* strains were tested, including *A. fumigatus*, *A. flavus*, *Aspergillus terreus*, and *Aspergillus niger*. No synergism was observed with VOR or ISA. Among 30 *Aspergillus* strains, 24 (80%) and 29 (97%) demonstrated synergism with OA/ITR and OA/POS, respectively (Fig. 1).

**TABLE 2:** *In vitro* drug sensitization results of OA combined with azoles against *Aspergillus* spp.^*a*^

Strains	MIC alone (µg/mL)					MIC combinations (µg/mL)			
	OA	ITR	VOR	ISA	POS	OA/ITR	OA/VOR	OA/ISA	OA/POS
*A. fumigatus*									
AF1	>8	4	0.125	0.5	1	2/1 (S)	16/0.125 (I)	16/0.5 (I)	2/0.125 (S)
AF2	>8	2	0.25	0.5	1	2/0.5 (S)	16/0.25 (I)	16/0.5 (I)	2/0.125 (S)
AF3	>8	2	0.125	0.25	1	2/0.5 (S)	16/0.125 (I)	16/0.25 (I)	2/0.063 (S)
AF4	>8	4	0.25	0.5	1	2/1 (S)	16/0.25 (I)	16/0.5 (I)	2/0.063 (S)
AF5	>8	4	1	1	2	2/0.5 (S)	16/1 (I)	16/1 (I)	2/0.063 (S)
AF6	>8	2	0.25	0.5	1	2/0.5 (S)	16/0.25 (I)	16/0.5 (I)	2/0.063 (S)
AF7	>8	2	0.25	0.5	1	2/1 (I)	16/0.25 (I)	16/0.5 (I)	4/0.063 (S)
AF8	>8	4	0.25	0.5	1	2/0.5 (S)	16/0.25 (I)	16/0.5 (I)	2/0.063 (S)
AF9	>8	2	4	8	1	2/0.5 (S)	16/4 (I)	16/8 (I)	4/0.063 (S)
AF10	>8	16	0.25	0.5	2	16/16 (I)	16/0.25 (I)	16/0.5 (I)	16/2 (I)
*A. flavus*									
AL1	>8	2	0.25	0.5	2	2/0.25 (S)	16/0.25 (I)	16/0.5 (I)	2/0.063 (S)
AL2	>8	2	0.25	0.5	1	2/0.5 (S)	16/0.25 (I)	16/0.5 (I)	2/0.063 (S)
AL3	>8	2	2	2	1	2/0.5 (S)	16/2 (I)	16/2 (I)	2/0.125 (S)
AL4	>8	2	0.25	0.5	1	2/0.5 (S)	16/0.25 (I)	16/0.5 (I)	2/0.125 (S)
AL5	>8	2	1	0.5	1	2/0.5 (S)	16/1 (I)	16/0.5 (I)	2/0.125 (S)
AL6	>8	2	0.25	0.5	1	2/0.5 (S)	16/0.25 (I)	16/0.5 (I)	2/0.125 (S)
AL7	>8	4	0.5	0.5	1	2/2 (I)	16/0.5 (I)	16/0.5 (I)	2/0.063 (S)
AL8	>8	2	0.25	0.5	1	2/0.5 (S)	0.125/0.125 (I)	16/0.5 (I)	4/0.25 (S)
AL9	>8	4	0.25	0.25	1	4/1 (S)	16/0.25 (I)	16/0.25 (I)	4/0.125 (S)
AL10	>8	4	0.125	0.5	1	2/2 (I)	16/0.125 (I)	16/0.5 (I)	4/0.125 (S)
AL11	>8	4	0.25	0.5	1	4/1 (S)	16/0.25 (I)	16/0.5 (I)	4/0.25 (S)
*A. terreus*									
AT1	>8	2	0.25	0.25	1	2/0.25 (S)	0.25/0.125 (I)	16/0.25 (I)	2/0.125 (S)
AT2	>8	4	0.25	0.5	1	4/1 (S)	16/0.25 (I)	16/0.5 (I)	4/0.125 (S)
AT3	>8	4	8	8	1	4/1 (S)	16/8 (I)	16/8 (I)	4/0.125 (S)
AT4	>8	4	0.25	0.5	1	4/0.5 (S)	16/0.25 (I)	16/0.5 (I)	4/0.063 (S)
AT5	>8	4	0.5	0.5	1	4/1 (S)	16/0.5 (I)	16/0.5 (I)	4/0.125 (S)
AT6	>8	4	0.25	0.5	1	4/1 (S)	16/0.25 (I)	16/0.5 (I)	4/0.125 (S)
*A. niger*									
AN1	>8	4	0.5	0.5	1	4/2 (I)	16/0.5 (I)	16/0.5 (I)	4/0.25 (S)
AN2	>8	8	0.25	0.5	1	2/2 (S)	16/0.25 (I)	16/0.5 (I)	4/0.25 (S)
AN3	>8	4	2	1	1	4/2 (I)	16/2 (I)	16/1 (I)	4/0.25 (S)
Quality control									
ATCC 204304	>8	2	1	0.25	1	2/0.5 (S)	16/1 (I)	16/0.25 (I)	2/0.125 (S)
ATCC 22019	>8	0.5	0.063	0.063	0.125	4/0.125 (S)	16/0.0625 (I)	16/0.0625 (I)	2/0.031 (S)

^*a*^ S, synergy (FICI ≤ 0.5); I, indifference (no interaction, FICI from >0.5 to ≤4). MICs were the concentrations that achieved 100% growth inhibition.

Further analysis of synergistic patterns revealed distinct results for the OA/ITR and OA/POS combinations. For the OA/ITR combination, among 10 *A*. *fumigatus* strains, 8 (80%) showed synergism; among 11 *A*. *flavus* strains, 9 (82%) demonstrated synergism; among 6 *A*. *terreus* strains, all 6 (100%) exhibited synergism; and among 3 *A*. *niger* strains, only 1 (33%) showed synergism. For the OA/POS combination, among 10 *A*. *fumigatus* strains, 9 (90%) showed synergism; among 11 *A*. *flavus* strains, all 11 (100%) demonstrated synergism; among 6 *A*. *terreus* strains, all 6 (100%) exhibited synergism; and among 3 *A*. *niger* strains, all 3 (100%) showed synergism. Analysis of different *Aspergillus* species showed that while the OA/ITR combination had varying levels of synergism across *A. fumigatus*, *A. flavus*, *A. terreus*, and *A. niger* strains (with 80%, 82%, 100%, and 33%, respectively), the OA/POS combination consistently demonstrated a higher efficacy, with 90%, 100%, 100%, and 100% of the same species’ strains showing synergism. These results show that the OA/POS combination outperforms the OA/ITR combination in terms of eliciting synergistic antifungal effects across multiple *Aspergillus* species.

### *In vitro* interactions between OA and azoles against *Candida* spp.

**Fig 2:**
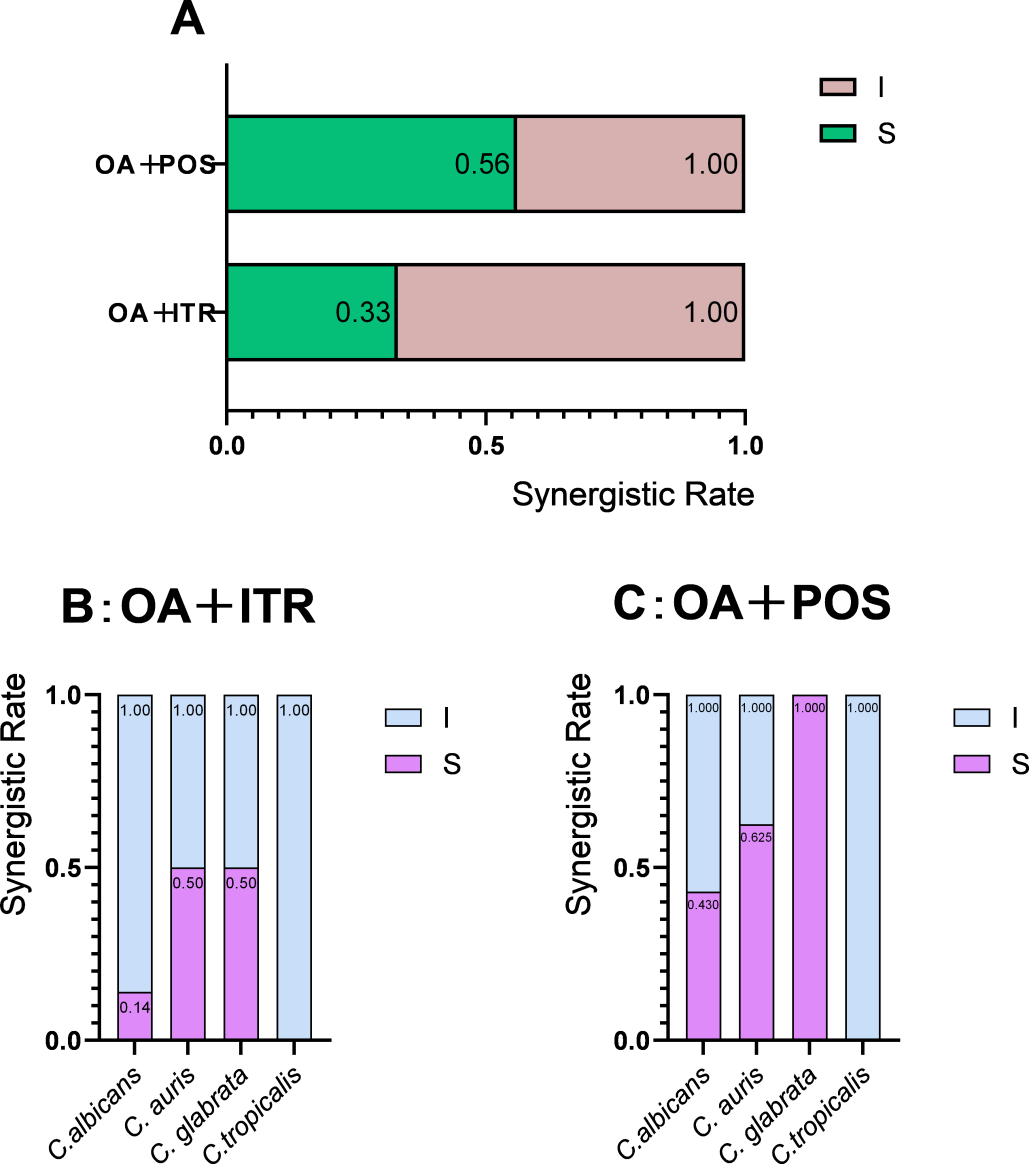
The synergistic rate of OA combined with azoles against *Candida* spp. (A) Interaction profile of OA in combination with ITR and POS against *Candida* spp.; (B) OA/ITR interaction profiles across *Candida* species; (C) OA/POS interaction profiles across *Candida* species. S, synergy (FICI ≤ 0.5); I, indifference (FICI from >0.5 to ≤4). The synergism rate was calculated by dividing the number of strains exhibiting synergism by the total number of strains tested.

OA alone demonstrated no antifungal activity against *Candida* spp. For synergistic combinations, the baseline MICs of azoles alone ranged from 0.25 to 2 µg/mL for ITR and 0.125–4 µg/mL for POS (Table 3). When combined with OA, the MIC ranges of azoles in synergistic combinations decreased to 0.063–0.25 µg/mL (ITR) and 0.031–0.125 µg/mL (POS), achieving maximum reductions of 8-fold and 127-fold, respectively. No synergism was observed with VOR or FLU. Among 18 *Candida* strains, 6 (33%) and 10 (56%) exhibited synergism with OA/ITR and OA/POS, respectively (Fig. 2). Species-specific analysis revealed synergism in 1/7 (14%) *C*. *albicans* strains with OA/ITR versus 3/7 (43%) with OA/POS; 4/8 (50%) *Candida auris* strains with OA/ITR versus 5/8 (62.5%) with OA/POS; 1/2 (50%) *C*. *glabrata* strains with OA/ITR versus 2/2 (100%) with OA/POS; and no synergism in the single *Candida tropicalis* strain tested. Consistent with findings in *Aspergillus*, OA combined with ITR and POS also demonstrated synergistic effects against *Candida* spp., with OA/POS showing the highest efficacy.

**TABLE 3:** *In vitro* drug sensitivity results of OA combined with azoles against *Candida* spp.^*a*^

Strains	MIC alone (µg/mL)					MIC combinations (µg/mL)			
	OA	ITR	VOR	FlU	POS	OA/ITR	OA/VOR	OA/FlU	OA/POS
*C. albicans*									
R1	>8	0.5	0.5	0.5	8	0.5/0.25 (I)	16/0.5 (I)	16/8 (I)	1/0.063 (S)
R2	>8	0.5	1	4	0.5	2/0.25 (I)	16/1 (I)	16/4 (I)	2/0.063 (S)
R3	>8	0.25	0.5	8	0.125	8/0.063 (S)	16/0.5 (I)	16/8 (I)	8/0.063 (I)
R4	>8	0.25	0.25	2	0.125	16/0.25 (I)	0.25/0.5 (I)	16/2 (I)	16/0.125 (I)
R5	>8	0.5	1	4	0.125	8/0.125 (I)	16/1 (I)	16/4 (I)	4/0.031 (S)
R6	>8	0.5	2	4	0.125	2/0.25 (I)	16/2 (I)	16/4 (I)	4/0.063 (I)
R7	>8	8	8	8	4	16/8 (I)	16/8 (I)	16/8 (I)	16/4 (I)
*C. auris*									
N1	>8	0.125	0.25	0.25	0.125	16/0.125 (I)	16/0.25 (I)	16/0.25 (I)	2/0.031 (S)
N2	>8	2	0.25	4	0.5	2/0.25 (S)	4/0.063 (S)	16/4 (I)	4/0.125 (S)
N3	>8	0.25	4	8	0.125	2/0.063 (S)	16/4 (I)	16/8 (I)	2/0.031 (S)
N4	>8	0.5	4	0.25	0.063	2/0.125 (S)	16/4 (I)	16/0.25 (I)	2/0.031 (I)
N5	>8	0.5	4	8	0.125	1/0.25 (I)	16/4 (I)	16/8 (I)	4/0.031 (S)
N6	>8	2	8	4	0.5	8/0.25 (I)	8/0.25 (I)	2/2 (I)	8/0.125 (I)
N7	>8	2	8	4	0.5	16/2 (I)	16/8 (I)	16/4 (I)	2/0.25 (I)
N8	>8	1	2	8	0.5	2/0.25 (S)	8/0.25 (I)	16/8 (I)	2/0.125 (S)
*C. glabrata*									
G1	>8	0.5	0.25	8	0.25	0.5/0.25 (I)	16/0.25 (I)	16/8 (I)	4/0.063 (S)
G2	>8	0.25	0.25	2	0.125	2/0.063 (S)	16/0.25 (I)	16/0.063 (I)	4/0.031 (S)
*C.tropicalis*									
T1	>8	0.25	1	0.125	1	16/0.25 (I)	16/1 (I)	16/1 (I)	1/0.063 (I)
Quality control									
ATCC204304	>8	2	1	0.25	1	2/0.5 (S)	16/1 (I)	16/0.25 (I)	2/0.125 (S)
ATCC22019	>8	0.5	0.063	0.063	0.125	4/0.125 (S)	16/0.0625 (I)	16/0.0625 (I)	2/0.031 (S)

^*a*^ S, synergy (FICI ≤ 0.5); I, indifference (no interaction, FICI from >0.5 to ≤4). MICs were the concentrations that achieved 100% growth inhibition.

### *In vitro* interactions between OA and azoles against *E. dermatitidis*

**Fig 3:**
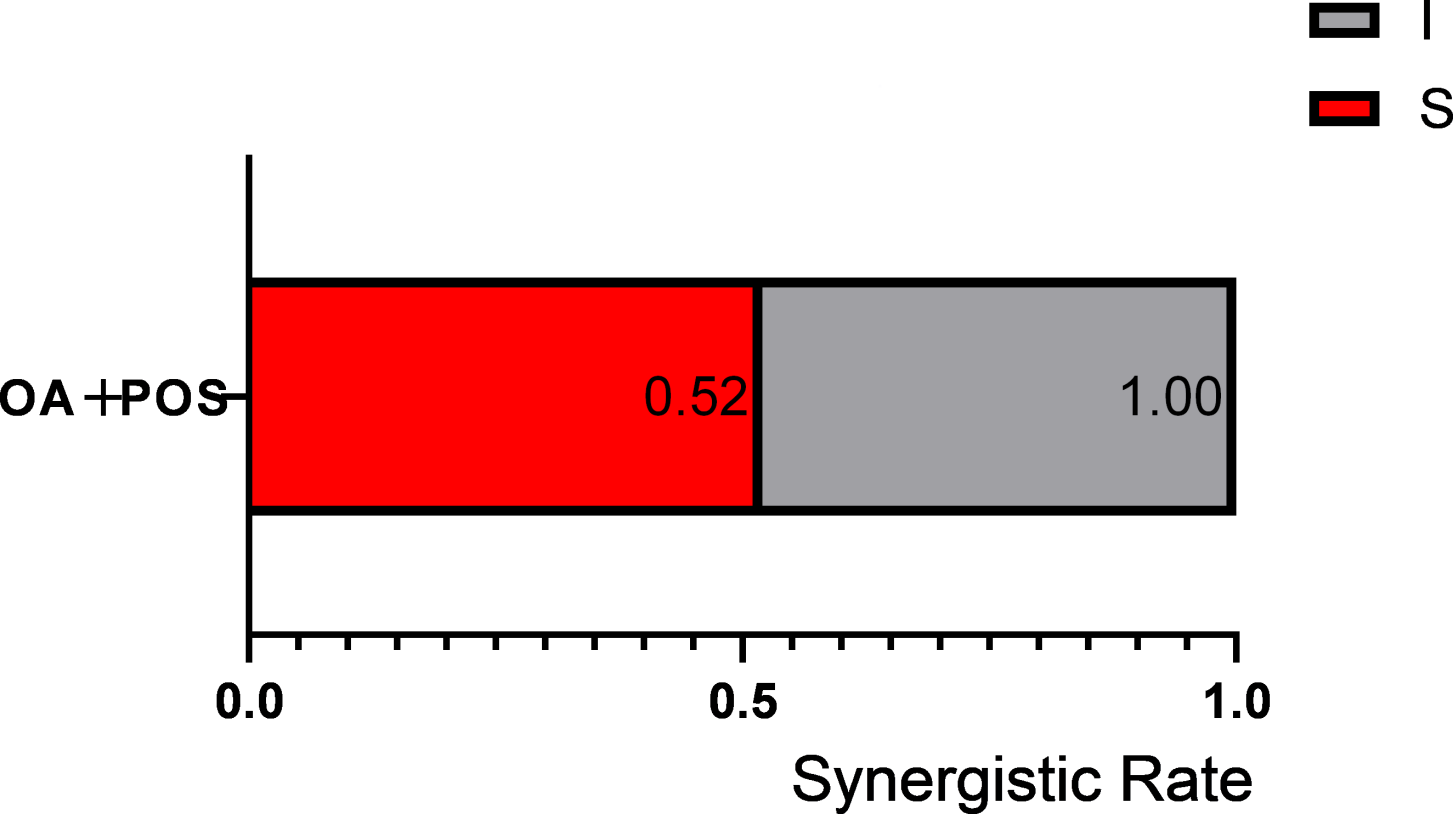
Interaction profile of OA in combination with POS against *E. dermatitidis*. S, synergy (FICI ≤ 0.5); I, indifference (FICI from >0.5 to ≤4). The synergism rate was calculated by dividing the number of strains exhibiting synergism by the total number of strains tested.

When used alone, OA failed to show any antifungal effect on *E. dermatitidis*. In contrast to other fungal genera, only the OA/POS combination demonstrated synergism among all tested regimens against *E. dermatitidis* (Table 4). For synergistic OA/POS combinations, the baseline MIC range of POS alone was 0.125–1 µg/mL, which decreased to 0.031–0.25 µg/mL when combined with OA, achieving a maximum MIC reduction of fourfold. Among 21 *E. dermatitidis* strains, 11 (52%) exhibited synergism with the OA/POS combination (Fig. 3).

**TABLE 4:** *In vitro* drug sensitization results of OA combined with azoles on *E. dermatitidis^a^*

Strains	MIC alone (µg/mL)					MIC combinations (µg/mL)			
	OA	ITR	VOR	ISA	POS	OA/ITR	OA/VOR	OA/ISA	OA/POS
BMU00028	>8	0.5	1	1	0.5	16/0.5 (I)	16/1 (I)	16/1 (I)	0.5/0.25 (I)
BMU00029	>8	0.5	1	1	0.5	16/0.5 (I)	16/1 (I)	16/1 (I)	1/0.25 (I)
BMU00030	>8	0.5	1	1	0.5	16/0.5 (I)	16/1 (I)	16/1 (I)	1/0.25 (I)
BMU00031	>8	1	1	2	0.5	16/1 (I)	16/1 (I)	16/2 (I)	1/0.25 (I)
BMU00034	>8	0.5	1	2	0.5	16/0.5 (I)	0.5/1 (I)	16/2 (I)	1/0.25 (I)
BMU00035	>8	0.5	0.5	0.5	0.25	1/0.25 (I)	16/0.5 (I)	16/0.5 (I)	1/0.125 (I)
BMU00036	>8	0.25	0.5	0.5	0.25	16/0.25 (I)	16/0.5 (I)	16/0.5 (I)	2/0.063 (S)
BMU00038	>8	1	1	2	0.5	0.25/0.5 (I)	16/1 (I)	16/2 (I)	1/0.125 (S)
BMU00039	>8	0.5	0.5	0.5	0.25	1/0.25 (I)	16/0.5 (I)	16/0.5 (I)	1/0.063 (S)
BMU00040	>8	0.05	0.25	0.25	0.125	1/0.125 (I)	16/0.25 (I)	16/0.25 (I)	1/0.031 (S)
BMU00041	>8	0.5	0.25	0.25	0.25	0.5/0.25 (I)	16/0.25 (I)	1/0.25 (I)	1/0.063 (S)
BMU00043	>8	0.5	1	1	0.5	16/0.5 (I)	16/1 (I)	16/1 (I)	1/0.125 (S)
NPRC 3.8.656	>8	0.5	0.5	0.5	1	16/0.5 (I)	16/1 (I)	16/0.5 (I)	1/0.25 (S)
NPRC 3.8.655	>8	0.5	0.5	1	0.5	16/0.5 (I)	0.125/1 (I)	16/1 (I)	1/0.125 (S)
NPRC 3.8.654	>8	1	1	1	0.5	0.25/0.5 (I)	16/1 (I)	16/1 (I)	1/0.125 (S)
NPRC 3.8.653	>8	0.5	0.5	0.25	0.125	1/0.25 (I)	16/0.5 (I)	16/0.25 (I)	1/0.063 (I)
NPRC 3.8.652	>8	0.5	0.5	0.5	0.5	1/0.25 (I)	16/0.5 (I)	16/0.5 (I)	0.5/0.125 (S)
109140	>8	1	1	1	0.5	0.5/0.5 (I)	16/1 (I)	16/1 (I)	1/0.125 (S)
109144	>8	8	4	8	4	16/16 (I)	16/4 (I)	0.5/4 (I)	16/4 (I)
109145	>8	1	0.5	1	0.25	0.5/0.5 (I)	16/0.5 (I)	16/1 (I)	1/0.125 (I)
109149	>8	4	0.5	4	1	16/4 (I)	16/0.5 (I)	16/4 (I)	16/1 (I)
Quality control									
ATCC204304	>8	2	1	0.25	1	2/0.5 (S)	16/1 (I)	16/0.25 (I)	2/0.125 (S)
ATCC22019	>8	0.5	0.063	0.063	0.125	4/0.125 (S)	16/0.063 (I)	16/0.063 (I)	2/0.031 (S)

^*a*^ S, synergy (FICI ≤ 0.5); I, indifference (no interaction, FICI from >0.5 to ≤4). MICs were the concentrations that achieved 100% growth inhibition.

### *In vitro* interactions between OA and azoles against *C. neoformans*

**Fig 4:**
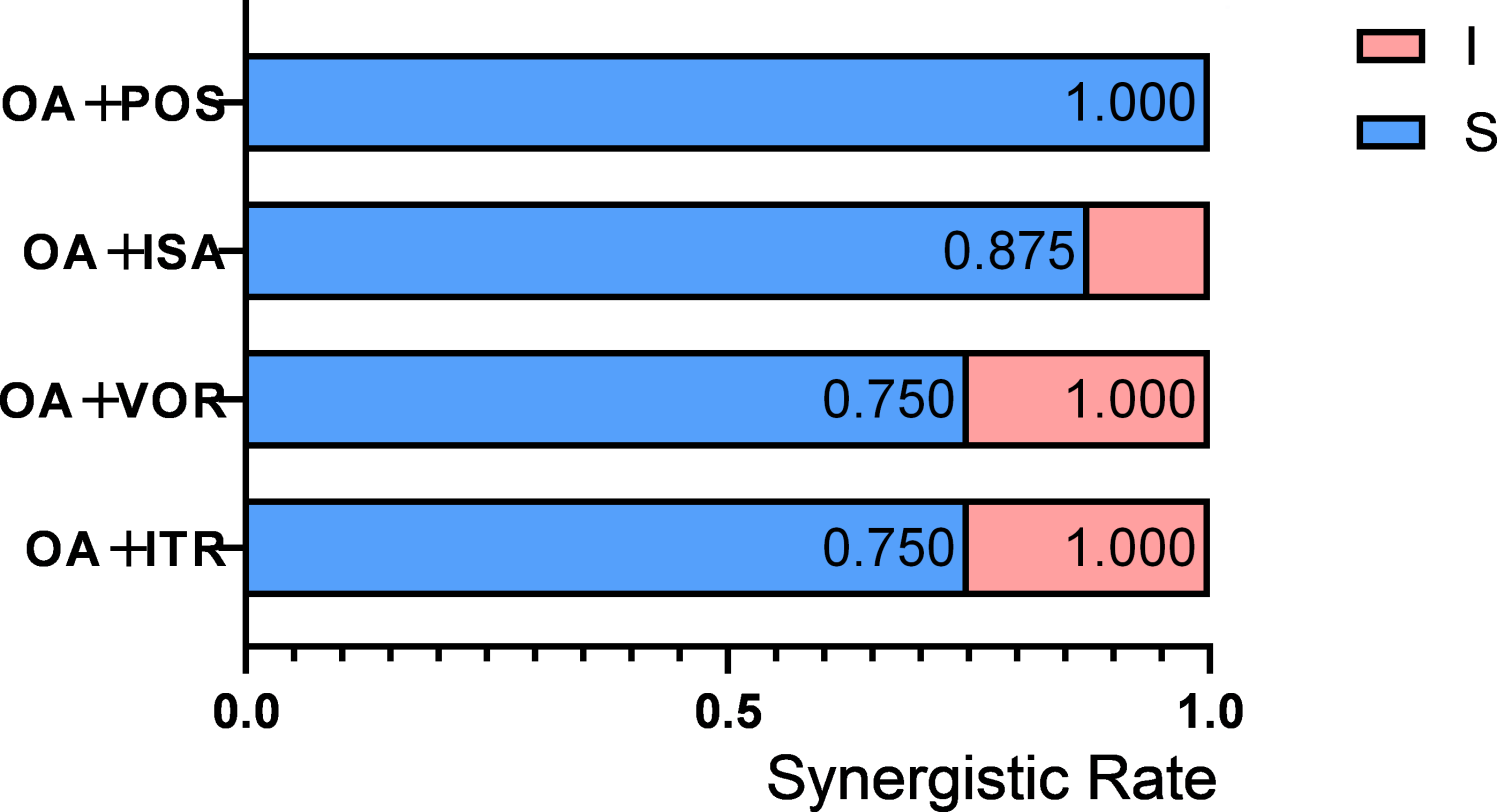
Interaction profiles of OA combined with ITR, VOR, ISA, and POS against *C. neoformans*. S, synergy (FICI ≤ 0.5); I, indifference (FICI from >0.5 to ≤4). The synergism rate was calculated by dividing the number of strains exhibiting synergism by the total number of strains tested.

OA, when used in isolation, did not display any inhibitory effect on C. *neoformans*. In contrast to other fungal genera, all four combinations of azoles with OA manifested synergistic effects (Table 5). Regarding the synergistic combinations, the baseline MIC ranges of azoles used independently were as follows: 2–4 µg/mL for ITR, 1–2 µg/mL for VOR, 1–4 µg/mL for ISA, and 1–2 µg/mL for POS. Upon combination with OA, these MIC ranges decreased. Specifically, for ITR, it decreased to 0.5–1 µg/mL; for VOR, it decreased to 0.25–0.5 µg/mL; for ISA, it decreased to 0.25–1 µg/mL; and for POS, it decreased to 0.125–0.5 µg/mL. The reductions in MIC values were fourfold for OA/ITR, OA/VOR, and OA/ISA and up to eightfold for OA/POS. Among the eight C. *neoformans* strains tested, synergistic outcomes were detected in six out of eight (75%) with OA/ITR, six out of eight (75%) with OA/VOR, seven out of eight (87.5%) with OA/ISA, and all eight out of eight (100%) with OA/POS (Fig. 4). Significantly, the OA/POS combination demonstrated the most remarkable synergistic effect.

**TABLE 5:** *In vitro* drug sensitivity results of OA combined with azoles against *C. neoformans^a^*

Strains	MIC alone (µg/mL)					MIC combinations (µg/mL)			
	OA	ITR	VOR	ISA	POS	OA/ITR	OA/VOR	OA/ISA	OA/POS
Y1	>8	2	1	1	2	2/0.5 (S)	4/0.25 (S)	4/0.25 (S)	4/0.25 (S)
Y2	>8	2	2	2	1	2/0.5 (S)	4/0.5 (S)	2/0.5 (S)	4/0.125 (S)
Y3	>8	2	2	4	2	4/0.5 (S)	1/0.5 (S)	0.25/1 (S)	4/0.25 (S)
Y4	>8	4	2	4	1	8/1 (I)	16/2 (I)	0.25/1 (S)	4/0.125 (S)
Y5	>8	4	2	2	2	0.125/1 (S)	4/0.5 (S)	0.25/0.5 (S)	4/0.5 (S)
Y6	>8	2	2	1	2	2/0.5 (S)	2/0.5 (S)	2/0.25 (S)	4/0.25 (S)
Y7	>8	2	2	2	1	8/0.5 (I)	16/2 (I)	16/2 (I)	4/0.25 (S)
Y8	>8	2	1	4	1	4/0.5 (S)	4/0.25 (S)	4/1 (S)	4/0.25 (S)
Quality control									
ATCC204304	>8	2	1	0.25	1	2/0.5 (S)	16/1 (I)	16/0.25 (I)	2/0.125 (S)
ATCC22019	>8	0.5	0.063	0.063	0.125	4/0.125 (S)	16/0.0625 (I)	16/0.0625 (I)	2/0.031 (S)

^*a*^ S, synergy (FICI ≤ 0.5); I, indifference (no interaction, FICI from >0.5 to ≤4). MICs were the concentrations that achieved 100% growth inhibition.

### Summary of *in vitro* interactions of OA combined with azoles against pathogenic fungi

**Fig 5:**
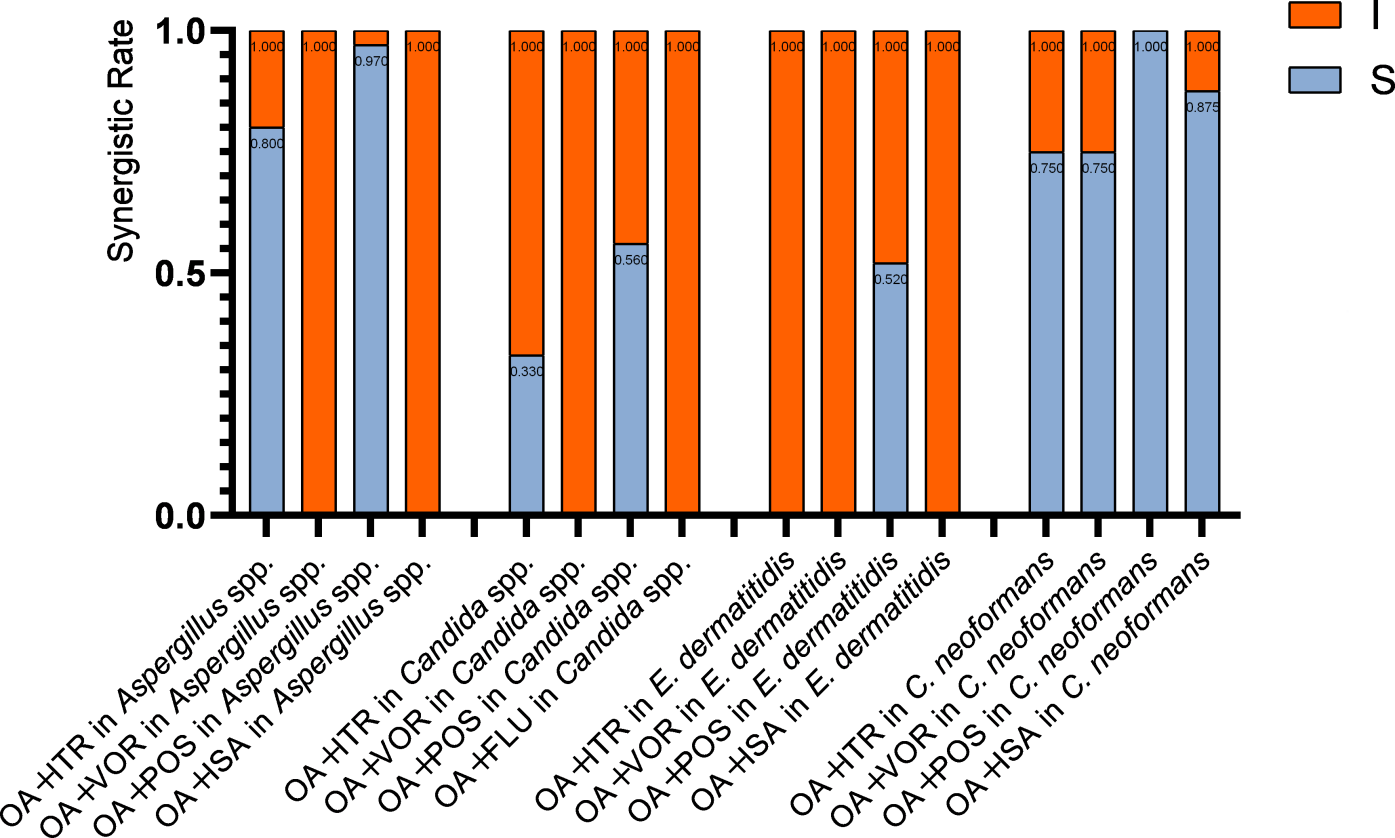
Species-specific summary of interactions between OA-azole combinations and pathogenic fungi.

**Fig 6:**
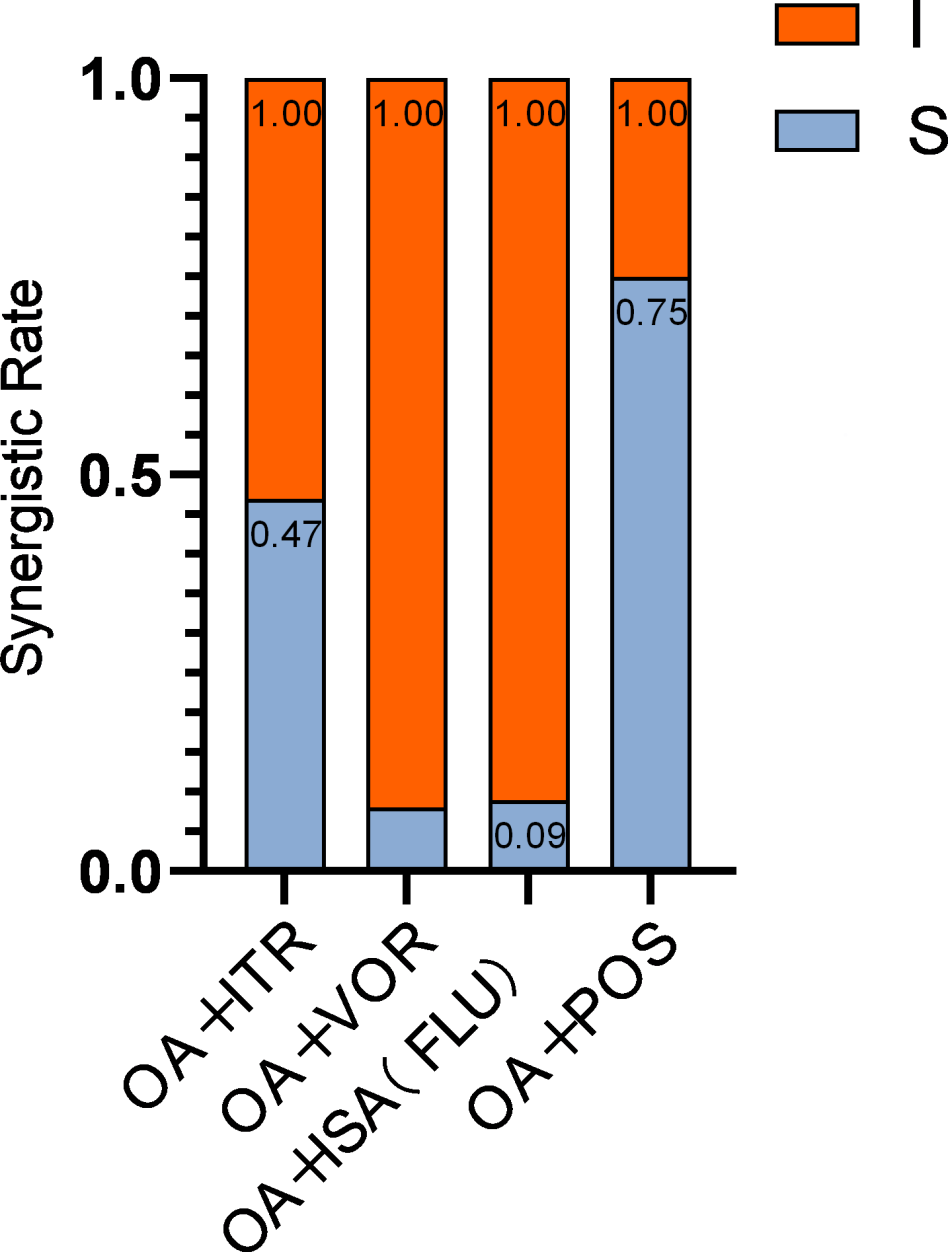
Azole-specific summary of interactions between OA-azole combinations and pathogenic fungi. S, synergy (FICI ≤ 0.5); I, indifference (FICI from >0.5 to ≤4). The synergism rate was calculated by dividing the number of strains exhibiting synergism by the total number of strains tested.

OA exhibited variable synergism with different azoles across pathogenic fungi (Table 6). Among 30 *Aspergillus* strains, 24 (80%) and 29 (97%) showed synergism with OA/ITR and OA/POS, respectively. For 18 *Candida* strains, synergism was observed in 6 (33%) with OA/ITR and 10 (56%) with OA/POS. Of 21 *E. dermatitidis* strains, 11 (52%) demonstrated synergism with OA/POS, while all 8 *C. neoformans* strains exhibited synergism across OA-azole combinations: 6/8 (75%) with OA/ITR and OA/VOR, 7/8 (87.5%) with OA/ISA, and 8/8 (100%) with OA/POS (Fig. 5). However, OA combined with VOR showed no synergism against *Aspergillus*, *Candida*, or *E. dermatitidis*; similarly, OA/ISA lacked activity against *Aspergillus* and *E. dermatitidis*. OA/ITR showed no synergism against *E. dermatitidis,* and OA/FLU showed no synergism against *Candida*, respectively. When categorized by azole type, the synergy rates of OA combined with ITR, VOR, ISA (FLU), and POS against all pathogenic fungi were 36/77 (47%), 6/77 (8%), 7/77 (9%), and 58/77 (75%), respectively (Fig. 6). Strikingly, *C. neoformans* was the sole pathogen that demonstrated universal synergism with all four OA-azole combinations.

**TABLE 6:** Summary of *in vitro* interaction results of OA combined with azoles against pathogenic fungi^*a*^

	ITR			VOR			ISA (FLU in *Candida* spp.)			POS		
	S	I	A	S	I	A	S	I	A	S	I	A
*Aspergillus* spp. (*n* = 30)	80%	20%	0%	0%	100%	0%	0%	100%	0%	97%	3%	0%
*Candida* spp. (*n* = 18)	33%	67%	0%	0%	100%	0%	0%	100%	0%	56%	44%	0%
*E. dermatitidis* (*n* = 21)	0%	100%	0%	0%	100%	0%	0%	100%	0%	52%	48%	0%
*C. neoformans* (*n* = 8)	75%	25%	0%	75%	25%	0%	87.5%	12.5%	0%	100%	0%	0%

^*a*^ S, synergy (FICI ≤ 0.5); I, indifference (FICI from >0.5 to ≤4); and A, antagonism (FICI > 4).

## DISCUSSION

To address the escalating challenge of antifungal resistance in pathogenic fungi, combination therapy has emerged as a strategic therapeutic approach. This study demonstrates that OA synergistically enhances azole efficacy against *Aspergillus* spp., *Candida* spp., *C. neoformans*, and *E. dermatitidis*. Notably, the OA/POS combination achieved the highest synergistic rate among all tested regimens against *Aspergillus* spp., while *C. neoformans* exhibited the broadest spectrum of synergistic interactions with multiple OA-azole combinations. For *E. dermatitidis*—a species regarded as potentially the most virulent causative agent of disseminated infections—we have demonstrated for the first time a synergistic effect between OA and POS against this pathogen (3). However, no synergism was detected for drug-resistant strains such as TR34 and TR46.

OA, a naturally occurring pentacyclic triterpenoid, exhibits multifaceted pharmacological activities, including hepatoprotective, anti-inflammatory, anticancer, and antifungal effects (27). A few studies reported that triterpenoids such as OA derivatives have antifungal effects (28). The antifungal activity of triterpenoids (non-OA) is associated with the inhibition of ergosterol biosynthesis and intracellular reactive oxygen species (ROS) accumulation (28). Azole antifungal drugs function as competitive inhibitors of lanosterol 14α-demethylase (CYP51), displacing lanosterol from the enzyme’s active site, thereby inducing ergosterol depletion and toxic methylated sterol intermediate accumulation, which ultimately leads to fungal growth arrest or cell death (29). Structurally, OA shares significant similarities with lanosterol, including a triterpenoid backbone, common biosynthetic intermediates (e.g., squalene), and dependence on CYP450-mediated catalysis during synthesis (30). These parallels suggest that OA may act as a competitive CYP51 inhibitor, blocking lanosterol access to the catalytic site and synergistically enhancing the antifungal efficacy of azoles (31). When combined with azoles, OA may potentiate antifungal effects by synergistically augmenting total intracellular ROS, ultimately leading to fungal growth inhibition. Additionally, OA induced cancer cell apoptosis by suppressing the mTOR signaling pathway and triggered autophagy in an AMPK-dependent manner *in vitro* (32). Remarkably, both mTOR and AMPK signaling pathways are highly conserved from yeast to mammals (33). This evolutionary conservation suggests that OA may augment the antifungal efficacy of azoles by activating apoptosis and autophagy through analogous TOR and AMPK regulatory mechanisms in fungi, thereby compensating for gaps in conventional azole-mediated antifungal action. However, this toxicity to cancer cells does not harm normal cells, such as hepatocytes (34).

Even at a low concentration (≤8 µg/mL), OA was able to exhibit significant synergy with azole drugs against these clinical isolation strains. Although OA alone at such a low dose was unable to kill fungi, synergistic effects were observed between OA and ITR or POS against *Aspergillus* spp., *Candida* spp., and *C. neoformans*. Notably, the OA/POS combination uniquely demonstrated synergism against *E. dermatitidis*. In contrast, OA paired with VOR or ISA showed no synergism against *Aspergillus* spp., *Candida* spp., or *E. dermatitidis* but displayed efficacy solely against *C. neoformans*, indicating that synergistic activity against *E. dermatitidis* is restricted to the OA/POS combination, whereas all four OA-azole pairings demonstrated synergism against *C. neoformans*. The diminished efficacy of VOR and ISA may stem from their short-tail structural configurations, which exhibit lower affinity for the CYP51 enzyme compared to long-tail antifungals (ITR and POS), resulting in inferior inhibition of ergosterol biosynthesis (35). Consistent with this rationale, POS, a derivative of ITR modified by elongating its side chain, achieves enhanced antifungal efficacy due to this structural alteration. The universal synergism of OA-azole combinations against *C. neoformans* likely relates to its unique structural characteristics, particularly its three canonical virulence factors—melanogenesis, polysaccharide capsule formation, and thermal adaptability (36)—with prior studies indicating that ursolic acid (an OA isomer) inhibits melanin and capsule production in *C. neoformans* strain H99 (37). This suggests that the pronounced synergism of OA against *C. neoformans* may derive from OA-mediated suppression of melanin and capsule biosynthesis. Collectively, these findings demonstrate that OA-azole combinations not only exhibit significant antifungal activity but also display broad-spectrum efficacy across diverse pathogenic fungi, with the observed mechanistic divergence in synergism patterns underscoring the structural specificity of azole derivatives and the biological uniqueness of target organisms.

This study reveals that the natural product OA synergistically enhances azole antifungal activity against *Aspergillus* spp., *Candida* spp., *C. neoformans*, and *E. dermatitidis*. Notably, we report for the first time that the OA/POS combination exerts synergistic effects against *E. dermatitidis*, while all OA-azole combinations universally synergized against *C. neoformans*. These findings provide a novel strategy for optimizing antifungal combination therapies, advance our understanding of fungal resistance mechanisms, and propose innovative approaches for clinical management of fungal infections. However, the current evidence remains confined to *in vitro* models. Future investigations should prioritize *in vivo* validation of OA-azole efficacy and address pharmacological challenges such as OA’s poor aqueous solubility through advanced drug delivery systems (e.g., lipid nanoparticles or cyclodextrin complexes) to enhance bioavailability, ultimately paving the way for clinical translation.

## Data Availability

The data sets used and/or analyzed during the current study are available from the corresponding author upon reasonable request.
